# Supplementary material for: Comparative multiomics analysis of cell physiological state after culture in a basket bioreactor
Source: Sci Rep. 2022 Nov 23;12:20161. doi: 10.1038/s41598-022-24687-4 (PMC9686226; doi:10.1038/s41598-022-24687-4)
Supplement: Supplementary file 1 — Supplementary Information 1. [file 41598_2022_24687_MOESM1_ESM.zip › raw data/Proteomic raw data/4.DiffExprAnalysis/EG.vs.CG/EG.vs.CG.heatmap.detail1.pdf]

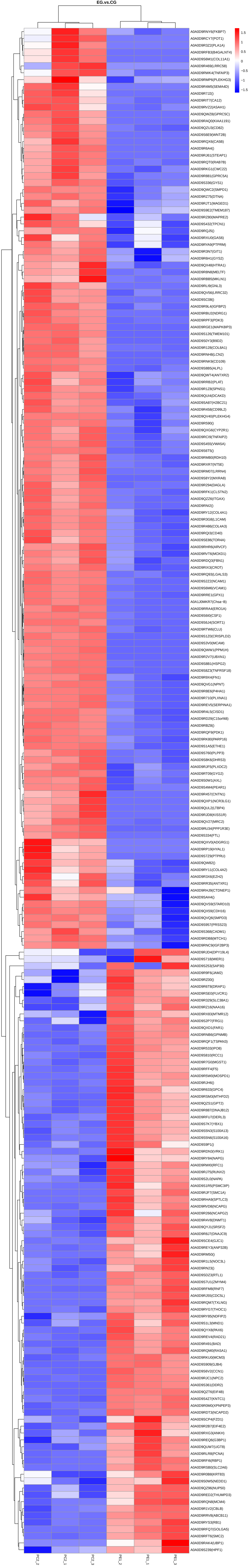
EG.vs.CG
1.5
1
0.5
0
−0.5
−1
−1.5
A0A0D9RNY6(FKBP7)
A0A0D9RCY7(POT1)
A0A0D9R3Z2(PLA1A)
A0A0D9RFB3(B4GALNT4)
A0A0D9S6M1(COL11A1)
A0A0D9R469(LRRC58)
A0A0D9RWK4(TNFAIP3)
A0A0D9RMP6(PLEKHG3)
A0A0D9R4M5(SEMA4D)
A0A0D9R7J2()
A0A0D9RIT7(CA12)
A0A0D9RVZ2(ASAH1)
A0A0D9QWZ8(GPRC5C)
A0A0D9RAQ0(KIAA1191)
A0A0D9QZU3(CD82)
A0A0D9S6E9(WNT2B)
A0A0D9RQX6(CA5B)
A0A0D9R6A4()
A0A0D9RJ61(STEAP1)
A0A0D9RQT0(RAB7B)
A0A0D9RKG1(CWC22)
A0A0D9R881(GPRC5A)
A0A0D9S336(GYS1)
A0A0D9QWC2(SMPD1)
A0A0D9RZ75(DTNA)
A0A0D9RJT1(MAGED1)
A0A0D9S9E2(TMEM187)
A0A0D9RZ80(MAPRE2)
A0A0D9S432(TPCN1)
A0A0D9RQJ5()
A0A0D9RXU0(GAS6)
A0A0D9RYA9(PTPRM)
A0A0D9R3N7(GIT1)
A0A0D9R6H1(GYS2)
A0A0D9QX48(HTRA1)
A0A0D9R8N8(MELTF)
A0A0D9RBB5(MKLN1)
A0A0D9RLI9(GNL3)
A0A0D9QV56(LRRC32)
A0A0D9SC08()
A0A0D9R9L4(IGFBP2)
A0A0D9RBU2(NDRG1)
A0A0D9RPF3(PDK3)
A0A0D9RGE1(MAPK8IP3)
A0A0D9S126(TMEM101)
A0A0D9S0Y3(B9D2)
A0A0D9R128(COL8A1)
A0A0D9RNH8(LCN2)
A0A0D9RNK9(CD109)
A0A0D9S8B5(ALPL)
A0A0D9QWT4(ANTXR2)
A0A0D9RRB2(PLAT)
A0A0D9R1Z8(SPNS1)
A0A0D9QUI4(DCAKD)
A0A0D9SA87(H2BC21)
A0A0D9R458(CD99L2)
A0A0D9QV40(PLEKHG4)
A0A0D9R590()
A0A0D9QXG6(CYP2R1)
A0A0D9RCI9(TNFAIP2)
A0A0D9S455(VWA5A)
A0A0D9S6T5()
A0A0D9RMB0(RDH10)
A0A0D9RXR7(NT5E)
A0A0D9RM07(LRRN4)
A0A0D9S8Y2(MXRA8)
A0A0D9R294(DAGLA)
A0A0D9RFK1(CLSTN2)
A0A0D9QZZ6(ITGAX)
A0A0D9RNI2()
A0A0D9RY12(COL4A1)
A0A0D9R3G8(L1CAM)
A0A0D9R486(COL4A3)
A0A0D9RQI3(CD40)
A0A0D9SE86(TOR4A)
A0A0D9RHR6(ARVCF)
A0A0D9RVT6(MOXD1)
A0A0D9RDQ3(FBN1)
A0A0D9RIX3(CROT)
A0A0D9RQ93(LGALS3)
A0A0D9S2Z2(NCAM1)
A0A0D9S6M6(VCAM1)
A0A0D9RRE1(GPX1)
A0A1J0MKR7(Chsa−B)
A0A0D9RRA4(ERO1A)
A0A0D9S6I0(CSF1)
A0A0D9S6J4(SORT1)
A0A0D9RTW6(CLU)
A0A0D9S1Z0(CRISPLD2)
A0A0D9S3V0(MCAM)
A0A0D9QWW1(PPM1H)
A0A0D9R2V7(UBXN1)
A0A0D9S8B1(HSPG2)
A0A0D9S8Z3(TNFRSF18)
A0A0D9R9X4(FN1)
A0A0D9QVG1(NPNT)
A0A0D9R8E6(P4HA1)
A0A0D9R710(PLXNA1)
A0A0D9REV5(SERPINA1)
A0A0D9R4L5(CISD1)
A0A0D9RD29(C15orf48)
A0A0D9RBZ8()
A0A0D9RQF9(PDK1)
A0A0D9RK80(PARP16)
A0A0D9S1A5(ETHE1)
A0A0D9S760(PLPP3)
A0A0D9S8K6(DHRS3)
A0A0D9RJF5(PLXDC2)
A0A0D9RT09(GYG2)
A0A0D9S0W1(AXL)
A0A0D9S4M4(PEAR1)
A0A0D9R457(CNTN1)
A0A0D9QXP1(NCR3LG1)
A0A0D9QUL2(LTBP4)
A0A0D9RJD8(KISS1R)
A0A0D9QV27(MRC2)
A0A0D9RU34(PPP1R3E)
A0A0D9S334(FTL)
A0A0D9QXV0(ADGRG1)
A0A0D9RP19(HYAL1)
A0A0D9S7Z9(PTPRU)
A0A0D9QW82()
A0A0D9RY11(COL4A2)
A0A0D9R3X6(EZH2)
A0A0D9RR35(ANTXR1)
A0A0D9RHJ9(CTDNEP1)
A0A0D9SAH4()
A0A0D9QVS9(STARD10)
A0A0D9QX59(CDH16)
A0A0D9QVQ6(SMPD3)
A0A0D9S957(PRSS23)
A0A0D9S368(CADM1)
A0A0D9RDB8(MTCH1)
A0A0D9RNC9(IGF2BP3)
A0A0D9RJD4(DPY19L4)
A0A0D9S718(MIER1)
A0A0D9S253(SAP30)
A0A0D9R9F6(JAM2)
A0A0D9RZ00()
A0A0D9R6T8(DRAP1)
A0A0D9RSE0(FLVCR1)
A0A0D9R329(SLC38A1)
A0A0D9RZ16(NAA16)
A0A0D9RX83(MTMR12)
A0A0D9S2P7(FRG1)
A0A0D9QXD1(FAR1)
A0A0D9RNB6(GPNMB)
A0A0D9RQF1(TSPAN3)
A0A0D9R533(IPO8)
A0A0D9S810(RCC1)
A0A0D9R7G0(MGST1)
A0A0D9RFF4(F5)
A0A0D9R5W0(MOSPD1)
A0A0D9RJH6()
A0A0D9R633(GPC4)
A0A0D9RSM3(MTHFD2)
A0A0D9QZS1(GPT2)
A0A0D9R887(DNAJB12)
A0A0D9RFU7(DERL3)
A0A0D9S7K7(YBX1)
A0A0D9S5N3(S100A13)
A0A0D9S5N6(S100A16)
A0A0D9S9P1()
A0A0D9RDN3(VRK1)
A0A0D9RY84(NAPG)
A0A0D9RWI0(RFC1)
A0A0D9RJ75(RUNX2)
A0A0D9S2L0(NAPA)
A0A0D9S1R5(PSMC3IP)
A0A0D9RJF7(SMC1A)
A0A0D9RHA9(SPTLC3)
A0A0D9RVD8(NCAPG)
A0A0D9R266(NCAPG2)
A0A0D9R4V8(DNMT1)
A0A0D9QYJ1(SRSF2)
A0A0D9R8J7(DNAJC9)
A0A0D9SCE4(GJC1)
A0A0D9REY3(ANP32B)
A0A0D9RM50()
A0A0D9R1L5(NOC3L)
A0A0D9RN23()
A0A0D9SDZ3(RTL1)
A0A0D9S7U1(ZMYM4)
A0A0D9RFM8(RNF7)
A0A0D9RJ55(CDC5L)
A0A0D9RQM7(TXLNG)
A0A0D9RYG7(THOC1)
A0A0D9RY85(NDFIP2)
A0A0D9S1L3(MND1)
A0A0D9QYX8(PAX6)
A0A0D9REV4(RAD21)
A0A0D9R491(BAD)
A0A0D9RQM0(RASA1)
A0A0D9RKU0(MCM3)
A0A0D9S909(GJB4)
A0A0D9S6V2(CCN1)
A0A0D9RJC1(NPC2)
A0A0D9S361(DDR2)
A0A0D9QZT6(EIF4B)
A0A0D9S4Z7(KNTC1)
A0A0D9R0M0(XPNPEP3)
A0A0D9RDT3(NCAPD2)
A0A0D9SCP4(FZD1)
A0A0D9R2B7(EIF4E2)
A0A0D9RXG3(ANKH)
A0A0D9REQ6(G3BP1)
A0A0D9QUW7(UGT8)
A0A0D9RLR8(PCNA)
A0A0D9RFI6(RBP1)
A0A0D9RSB0(SLC2A6)
A0A0D9R0B8(KRT83)
A0A0D9S0W5(NEDD1)
A0A0D9QZ96(NUP50)
A0A0D9RED2(THUMPD3)
A0A0D9RQN8(MCM4)
A0A0D9R1V2(CBLB)
A0A0D9RRV8(ABCB11)
A0A0D9RYS3(RB1)
A0A0D9RFQ7(GOLGA5)
A0A0D9RFT6(SMC2)
A0A0D9RAK4(UBP1)
A0A0D9S239(HPF1)
PC2_2
PC2_1
PC2_3
PE1_2
PE1_1
PE1_3
